# Colonic Adenocarcinoma with Plasmacytoid Feature: Histopathology and Molecular Characteristics of a Rare Neoplasm with an Unusual Presentation

**DOI:** 10.1155/2022/2640456

**Published:** 2022-02-08

**Authors:** Noor Marji, Jasrerman Dhillon, Gregory Y. Lauwers, Sebastian Feuerlein, Reza Nikfar, Monica Chatwal, Aram Vosoughi

**Affiliations:** ^1^Department of Pathology, H Lee Moffitt Cancer Center and Research Institute, 12902 USF Magnolia Drive, Tampa, FL 33612, USA; ^2^Department of Radiology, H Lee Moffitt Cancer Center and Research Institute, Tampa, FL, USA; ^3^Department of Genitourinary Oncology, H Lee Moffitt Cancer Center and Research Institute, Tampa, FL, USA

## Abstract

Colorectal carcinoma with noncohesive tumor cells has been described in tumors with signet ring cells (mucinous adenocarcinoma and signet ring cell adenocarcinoma) and rhabdoid feature (carcinoma with sarcomatoid component). Cases of carcinoma with plasmacytoid morphology are rare in the gastrointestinal tract, and a single case of plasmacytoid colorectal carcinoma has been reported. We report the case of a 37-year-old woman who presented with urinary symptoms, hematuria, and abdominal pain. Imaging studies showed segmental sigmoid wall thickening with pericolic infiltration and focal bladder wall thickening. The cystoscopy with transurethral resection of bladder tumor revealed muscle invasion, dis-cohesive carcinoma with plasmacytoid morphology, which was initially misdiagnosed as the plasmacytoid urothelial carcinoma. Immunohistochemical stains showed the tumor cells to be positive for CDX2, CK20, and SATB2 and negative for p63, GATA3, CK7, and Uroplakin II, indicating the colorectal origin of the tumor. The subsequent colonic wall biopsy showed the same tumor. Molecular studies identified BRAF V600E, SMAD4, and p53 mutations associated with aggressive colorectal adenocarcinoma with mucinous/signet ring cell features. Further whole-exome sequencing and whole transcriptome analysis confirmed the colorectal origin of the tumor. This rare colorectal adenocarcinoma with the plasmacytoid feature may represent the signet ring cell adenocarcinoma lacking extracellular mucin or intracellular vacuole. Diagnosis of this rare histological subtype of colorectal carcinoma is important, particularly in the unusual presentation of this aggressive tumor.

## 1. Introduction

Colorectal cancer (CRC) is one of most common cancers worldwide, and different histological variants of colorectal carcinoma are described based on the recent WHO classification of tumors [1]. Among this spectrum of CRC, noncohesive tumor cells have been described in tumors with signet ring cells (mucinous adenocarcinoma and signet ring cell carcinoma) and rhabdoid feature (carcinoma with sarcomatoid component). Cases of carcinoma with plasmacytoid morphology are rare in the GI tract and have been predominantly described in the stomach [2, 3]. To date, a single case of CRC with plasmacytoid features without molecular studies has been reported [3]. When initially presenting with bladder extension and urinary symptoms, establishing a diagnosis of CRC can be challenging and even more so in cases with plasmacytoid features as it should be differentiated from rare, aggressive plasmacytoid urothelial carcinoma (UC) [4, 5]. We report herein our experience with a very rare case of plasmacytoid colorectal carcinoma that caused significant diagnostic pitfall by its unusual clinical, radiologic, and pathologic presentations.

## 2. Case Presentation

**Figure 1:**
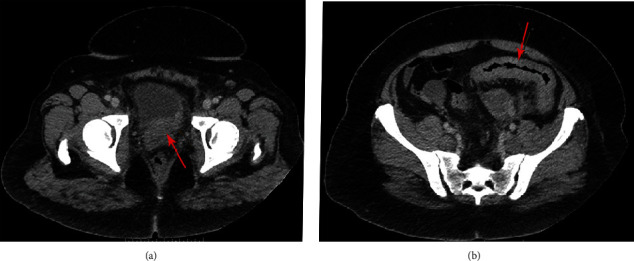
Axial abdominal computed tomography (CT): urinary bladder with thickening of the left posterior wall (a); midportion of sigmoid colon with wall thickening and pericolic infiltration suggestive of acute-subacute segmental colitis (b).

A 37-year-old woman with no significant past medical history presented with abdominal pain, hematuria, and urinary symptoms. Abdominal CT scan showed wall thickening of the midportion of the sigmoid with pericolic infiltration suggestive of acute–subacute segmental colitis with extensive abdominal and pelvic lymphadenopathy. Mild irregular wall thickening of the left posterior bladder wall raised the suspicion for primary bladder cancer (Figure 1). There were multiple bladder nodules on cystoscopy, and transurethral resection of bladder tumor (TURBT) revealed dis-cohesive malignant neoplasm with plasmacytoid features. Fine needle aspiration (FNA) of the peripancreatic lymph node showed metastatic carcinoma with similar morphology. Subsequently, the patient underwent sigmoid colon biopsy due to colonic obstruction, which showed a malignant process with the same morphology. Unfortunately, the patient died within five weeks of diagnosis before receiving any systemic therapy.

## 3. Histopathology

**Figure 2:**
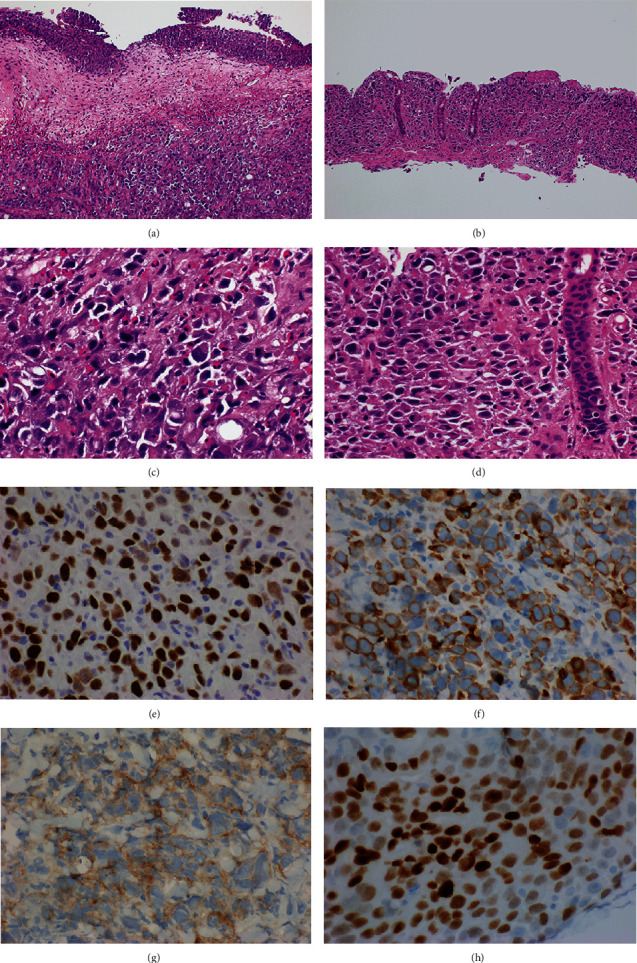
Histologic and immunohistochemical features of plasmacytoid colorectal carcinoma (CRC): plasmacytoid CRC diffusely invades bladder wall with benign overlying urothelium (10x, 40x) (a, c); plasmacytoid CRC invades colonic mucosa, and rare signet ring cells are present (10x, 40x) (b, d); plasmacytoid CRC with immunoreactivity for SATB2 (e); CK20 (f); CD138 (g); CDX2 (h).

Histologic evaluation of TURBT showed invasive dis-cohesive malignant cells with plasmacytoid features characterized by epithelioid cells with pale to eosinophilic cytoplasm and eccentrically placed enlarged nuclei with small nucleoli. The neoplastic cells invaded into the muscularis propria; however, the overlying urothelium showed no evidence of urothelial dysplasia or carcinoma in situ. The histologic evaluation of the colon and peripancreatic lymph node biopsies showed a similar histomorphology of invasive dis-cohesive carcinoma with plasmacytoid features (Figure 2). The differential diagnosis based on the histopathology includes plasmacytoid UC, metastatic breast lobular carcinoma, signet ring cell carcinoma of the GI tract (particularly gastric), plasmacytoma, and lymphoma [6]. Immunohistochemical stains performed on the biopsy specimens showed that tumor cells were negative for urothelial carcinoma markers (GATA3, uroplakin II, p63, CK7, CK5/6, CK-903) [7], breast carcinoma markers (ER and GATA3) [6], CD45, and neuroendocrine markers (synaptophysin, chromogranin). Alternatively, the tumor cells were positive for CK20, CDX2, CK8/18, and SATB2, consistent with colonic origin [8]. In addition, the tumor was also positive for CD138, which is reported in plasmacytoid UCs and the single reported plasmacytoid CRC. Tumor expressed E-Cadherin, which is mostly negative in plasmacytoid UC [9]. Immunostaining and next-generation sequencing (NGS) showed proficient DNA mismatch repair.

## 4. Molecular Studies

Targeted NGS, including 170 genes, showed BRAF p.V600E mutation, two p53 mutations (p.R248W and p.R175H), and SMAD4 p.R361C. These mutations are most commonly seen in colorectal cancers in AACR GENIE cases (one of the largest cancer genomic data sets) [10]. BRAF p.V600E mutated colorectal carcinoma frequently demonstrates invasive characteristics and histologic features such as lymphatic invasion, extracellular mucin, and signet ring cells [11]. Colorectal carcinoma with SMAD4 p.R361C more often presents with higher stage, tumor deposits, nodal metastasis, and mucinous features [12]. Two p53 mutations (p.R248W and p.R175H) increase tumor treatment resistance, invasion, and metastasis of CRC by increasing the stem cell population [13]. Also, CDH1 mutation, which is frequently reported in plasmacytoid UC, was not identified [9]. Furthermore, we applied a Genomic Prevalence Score (MI GPSai™) using whole-exome sequencing and whole transcriptome (RNA) analysis coupled with machine learning to confirm tumor origin [14]. MI GPSai predicts the tumor type with an accuracy of 94% by matching tumor molecular signature across 21 cancer types. The MI GPSai showed that the prevalence of tumor molecular signature is 96% among CRC, while its prevalence is less than 1% among UC.

## 5. Discussion

Colorectal cancers (CRC) with noncohesive tumor cells have been described in some histopathologic subtypes of colorectal carcinoma, including signet-ring cell carcinoma, mucinous adenocarcinoma (with signet ring cells), and carcinoma with sarcomatoid components (with rhabdoid cells). Gastrointestinal tract carcinoma with the plasmacytoid feature is a rare tumor that has been mainly described in the stomach, with a single reported case of plasmacytoid CRC [2, 3]. Diagnosis of CRC, initially presenting with bladder invasion, can be challenging, particularly in a very rare histologic subtype of CRC resembling well-known aggressive plasmacytoid UC. The clinical, radiologic presentation and plasmacytoid morphology of the tumor presented in this manuscript initially resulted in a misdiagnosis of plasmacytoid UC [6]; however, immunostaining was consistent with CRC. Although some CRC markers such as CK20 and CDX2 may be seen in UC, no expression of the more common UC markers such as CK-903, CK7, GATA3, and p63 was very unusual [6, 7]. A subsequent biopsy showed the same tumor involving the colonic wall. Moreover, whole-exome sequencing and whole transcriptome analysis (MI GPSai™) confirmed the CRC origin of cancer. Targeted NGS showed significant mutations, including BRAF p.V600E, SMAD4 p.R361C, and p53 p.R248W and p.R175H, all predominantly seen in aggressive CRC, often with mucinous/signet ring cell features [11–13]. Therefore, this rare plasmacytoid colorectal carcinoma may represent the signet ring cell CRC lacking extracellular mucin or intracellular vacuole. Signet ring adenocarcinoma has a low prevalence rate, approximately 1% of CRC patients, often present at an advanced stage and has been associated with a poor prognosis compared with common colonic adenocarcinoma [15]. This second reported case of plasmacytoid CRC indicates that comprehensive immunostaining and molecular studies maybe essential to diagnose this rare entity given its unusual presentation due to high invasive/metastatic potential.

## Data Availability

The datasets generated during and/or analyzed during the current study are available from the corresponding author on reasonable request.

## References

[1] Nagtegaal I. D., Odze R. D., Klimstra D. (2020). The 2019 WHO classification of tumours of the digestive system. *Histopathology*.

[2] Terada T. (2012). Primary CD138-positive poorly cohesive adenocarcinoma of the stomach whose carcinoma cells resemble plasma cells (plasmacytoid adenocarcinoma of the stomach). *Journal of Gastrointestinal Cancer*.

[3] Kovacs Z., Gurzu S., Molnar C. (2019). Gastrointestinal carcinoma with plasmacytoid morphology: positivity for c-MET, arylsulfatase, and markers of epithelial-mesenchymal transition, as indicators of aggressivity. *Journal of Oncology*.

[4] Sood S., Paner G. P. (2019). Plasmacytoid urothelial carcinoma: an unusual variant that warrants aggressive management and critical distinction on transurethral resections. *Archives of Pathology & Laboratory Medicine*.

[5] Nigwekar P., Tamboli P., Amin M. B., Osunkoya A. O., Ben-Dor D., Amin M. B. (2009). Plasmacytoid urothelial carcinoma. *The American Journal of Surgical Pathology*.

[6] Borhan W. M., Cimino-Mathews A. M., Montgomery E. A., Epstein J. I. (2017). Immunohistochemical differentiation of plasmacytoid urothelial carcinoma from secondary carcinoma involvement of the bladder. *The American Journal of Surgical Pathology*.

[7] Paner G. P., Annaiah C., Gulmann C. (2014). Immunohistochemical evaluation of novel and traditional markers associated with urothelial differentiation in a spectrum of variants of urothelial carcinoma of the urinary bladder. *Human Pathology*.

[8] Dragomir A., de Wit M., Johansson C., Uhlen M., Pontén F. (2014). The role of SATB2 as a diagnostic marker for tumors of colorectal origin: results of a pathology-based clinical prospective study. *American Journal of Clinical Pathology*.

[9] al-Ahmadie H. A., Iyer G., Lee B. H. (2016). Frequent somatic _*CDH1*_ loss-of-function mutations in plasmacytoid variant bladder cancer. *Nature Genetics*.

[10] AACR Project GENIE (2017). AACR Project GENIE: powering precision medicine through an international consortium. *Cancer Discovery*.

[11] Pai R. K., Jayachandran P., Koong A. C. (2012). BRAF-mutated, microsatellite-stable adenocarcinoma of the proximal colon: an aggressive adenocarcinoma with poor survival, mucinous differentiation, and adverse morphologic features. *The American Journal of Surgical Pathology*.

[12] Liao X., Hao Y., Zhang X. (2019). Clinicopathological characterization of SMAD4-mutated intestinal adenocarcinomas: a case-control study. *PLoS One*.

[13] Solomon H., Dinowitz N., Pateras I. S. (2018). Mutant p53 gain of function underlies high expression levels of colorectal cancer stem cells markers. *Oncogene*.

[14] Abraham J., Heimberger A. B., Marshall J. (2021). Machine learning analysis using 77,044 genomic and transcriptomic profiles to accurately predict tumor type. *Translational Oncology*.

[15] Hyngstrom J. R., Hu C. Y., Xing Y. (2012). Clinicopathology and outcomes for mucinous and signet ring colorectal adenocarcinoma: analysis from the National Cancer Data Base. *Annals of Surgical Oncology*.

